# Supplementary material for: On the cross-population generalizability of gene expression prediction models
Source: PLoS Genet. 2020 Aug 14;16(8):e1008927. doi: 10.1371/journal.pgen.1008927 (PMC7449671; doi:10.1371/journal.pgen.1008927)
Supplement: S12 Table — (DOCX) [file pgen.1008927.s012.docx]

| **Train-Test** | **YRI proportion** | **Power** | **95% CI** | **Train-Test** | **YRI proportion** | **Power** | **95% CI** |
| --- | --- | --- | --- | --- | --- | --- | --- |
| AD to CEU | 0.0 | 0.92 | 0.867 - 0.973 | CEU to YRI | 0.0 | 0.59 | 0.493 - 0.687 |
| AD to CEU | 0.1 | 0.92 | 0.867 - 0.973 | CEU to YRI | 0.1 | 0.57 | 0.472 - 0.668 |
| AD to CEU | 0.2 | 0.92 | 0.867 - 0.973 | CEU to YRI | 0.2 | 0.56 | 0.462 - 0.658 |
| AD to CEU | 0.3 | 0.93 | 0.880 - 0.980 | CEU to YRI | 0.3 | 0.56 | 0.462 - 0.658 |
| AD to CEU | 0.4 | 0.95 | 0.907 - 0.993 | CEU to YRI | 0.4 | 0.56 | 0.462 - 0.658 |
| AD to CEU | 0.5 | 0.92 | 0.867 - 0.973 | CEU to YRI | 0.5 | 0.56 | 0.462 - 0.658 |
| AD to CEU | 0.6 | 0.92 | 0.867 - 0.973 | CEU to YRI | 0.6 | 0.56 | 0.462 - 0.658 |
| AD to CEU | 0.7 | 0.93 | 0.880 - 0.980 | CEU to YRI | 0.7 | 0.56 | 0.462 - 0.658 |
| AD to CEU | 0.8 | 0.90 | 0.841 - 0.959 | CEU to YRI | 0.8 | 0.57 | 0.472 - 0.668 |
| AD to CEU | 0.9 | 0.89 | 0.828 - 0.952 | CEU to YRI | 0.9 | 0.56 | 0.462 - 0.658 |
| AD to CEU | 1.0 | 0.92 | 0.867 - 0.973 | CEU to YRI | 1.0 | 0.60 | 0.503 - 0.697 |
| AD to YRI | 0.0 | 0.88 | 0.816 - 0.944 | YRI to AD | 0.0 | 0.98 | 0.952 - 1.008 |
| AD to YRI | 0.1 | 0.95 | 0.907 - 0.993 | YRI to AD | 0.1 | 0.98 | 0.952 - 1.008 |
| AD to YRI | 0.2 | 0.95 | 0.907 - 0.993 | YRI to AD | 0.2 | 0.97 | 0.936 - 1.004 |
| AD to YRI | 0.3 | 0.94 | 0.893 - 0.987 | YRI to AD | 0.3 | 1.00 | 1.00 - 1.00 |
| AD to YRI | 0.4 | 0.95 | 0.907 - 0.993 | YRI to AD | 0.4 | 0.97 | 0.936 - 1.004 |
| AD to YRI | 0.5 | 0.94 | 0.893 - 0.987 | YRI to AD | 0.5 | 0.99 | 0.970 - 1.010 |
| AD to YRI | 0.6 | 0.94 | 0.893 - 0.987 | YRI to AD | 0.6 | 0.96 | 0.921 - 0.999 |
| AD to YRI | 0.7 | 0.94 | 0.893 - 0.987 | YRI to AD | 0.7 | 0.97 | 0.936 - 1.004 |
| AD to YRI | 0.8 | 0.93 | 0.880 - 0.980 | YRI to AD | 0.8 | 0.99 | 0.970 - 1.010 |
| AD to YRI | 0.9 | 0.94 | 0.893 - 0.987 | YRI to AD | 0.9 | 1.00 | 1.00 - 1.00 |
| AD to YRI | 1.0 | 0.95 | 0.907 - 0.993 | YRI to AD | 1.0 | 0.98 | 0.952 - 1.008 |
| CEU to AD | 0.0 | 0.99 | 0.970 - 1.010 | YRI to CEU | 0.0 | 0.63 | 0.535 - 0.725 |
| CEU to AD | 0.1 | 0.98 | 0.952 - 1.008 | YRI to CEU | 0.1 | 0.61 | 0.514 - 0.706 |
| CEU to AD | 0.2 | 0.99 | 0.970 - 1.010 | YRI to CEU | 0.2 | 0.59 | 0.493 - 0.687 |
| CEU to AD | 0.3 | 0.96 | 0.921 - 0.999 | YRI to CEU | 0.3 | 0.59 | 0.493 - 0.687 |
| CEU to AD | 0.4 | 0.97 | 0.936 - 1.004 | YRI to CEU | 0.4 | 0.59 | 0.493 - 0.687 |
| CEU to AD | 0.5 | 0.98 | 0.952 - 1.008 | YRI to CEU | 0.5 | 0.59 | 0.493 - 0.687 |
| CEU to AD | 0.6 | 0.97 | 0.936 - 1.004 | YRI to CEU | 0.6 | 0.58 | 0.483 - 0.677 |
| CEU to AD | 0.7 | 0.98 | 0.952 - 1.008 | YRI to CEU | 0.7 | 0.58 | 0.483 - 0.677 |
| CEU to AD | 0.8 | 0.99 | 0.970 - 1.010 | YRI to CEU | 0.8 | 0.58 | 0.483 - 0.677 |
| CEU to AD | 0.9 | 0.92 | 0.867 - 0.973 | YRI to CEU | 0.9 | 0.59 | 0.493 - 0.687 |
| CEU to AD | 1.0 | 0.95 | 0.907 - 0.993 | YRI to CEU | 1.0 | 0.63 | 0.535 - 0.725 |

Supplementary Table 12: Power estimates and 95% confidence intervals for each train-test scenario (Train-Test) and each proportion of YRI (YRI proportion) corresponding to the right panel of Figure 7 for effect size 0.01 (h^2^ = 0.58).
