# Supplementary figures and images for: On the cross-population generalizability of gene expression prediction models
Source: PLoS Genet. 2020 Aug 14;16(8):e1008927. doi: 10.1371/journal.pgen.1008927 (PMC7449671; doi:10.1371/journal.pgen.1008927)

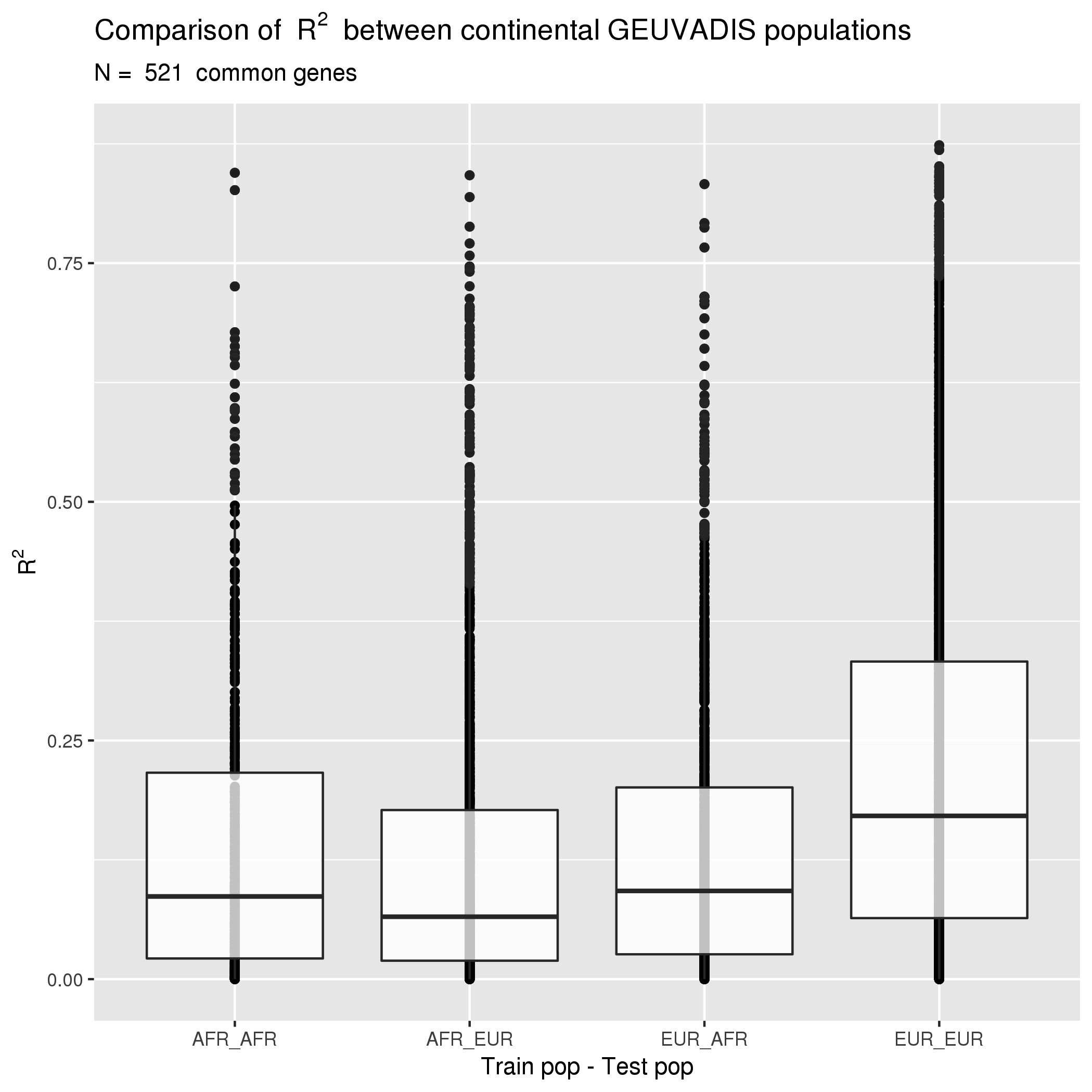

Supplement: S17 Fig — Predicting into and from AFR produces consistently lower R2 than predicting within EUR, suggesting a potential decrease in prediction accuracy when predicting across continental population groups. (TIFF) [file pgen.1008927.s029.tiff]

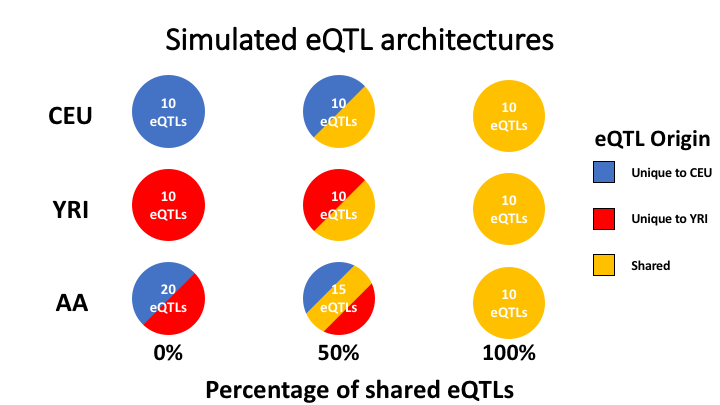

Supplement: S19 Fig — Blue encodes eQTLs specific to CEU; red encodes eQTLs specific to YRI; and gold encodes eQTLs shared between CEU and YRI. Models for CEU and YRI always had k eQTLs. AA always inherited all eQTLs from the ancestral populations. Consequently, the number of eQTLs in AA varied depending on how many eQTLs CEU and YRI shared. (TIFF) [file pgen.1008927.s031.tiff]
