# Supplementary material for: On the cross-population generalizability of gene expression prediction models
Source: PLoS Genet. 2020 Aug 14;16(8):e1008927. doi: 10.1371/journal.pgen.1008927 (PMC7449671; doi:10.1371/journal.pgen.1008927)
Supplement: Attachment — Submitted filename: PLOS Genetics Response to Reviewers current draft.pdf [file pgen.1008927.s036.pdf]

Reviewer #1: Keys et al. use gene expression prediction models publicly available in PredictDB from multiple cohorts (DGN, GTEx, MESA) to predict gene expression in SAGE (n=39), an African American pediatric asthma cohort with genome-wide genotypes and whole blood RNA-Seq available. They assess predictive performance by comparing predicted expression to observed. They go on to build prediction models in GEUVADIS and test them within and between EUR and AFR populations within GEUVADIS. They also generate simulated African American data and show predictive performance and TWAS power increases with increased shared eQTL genetic architecture.

I have reviewed this paper previously at another journal and while the authors have added TWAS simulations and addressed most of my concerns, a major one remains.

We thank the reviewer for their prior reading of our manuscript and for noting the changes made in response to previous reviews. We address any remaining concerns below.

Since prediction performance in SAGE is poor across all PredictDB models tested, even among the best-predicted genes (Fig 3), is it the best cohort to use to address the question of cross-population generalizability, especially when larger datasets (GEUVADIS) are available? The authors should address whether a test set of 39 individuals is a suitable sample size for reliable estimates of performance.

We are sympathetic to this concern. We highlight SAGE as a candidate study that may represent a reasonable opportunity for PrediXcan-like approaches. However our manuscript relies on numerous other analyses of the PrediXcan weights or deriving novel weights, such as in our GEUVADIS analyses. Many research groups currently have similarly small quantities of expression data. Our own concern about the limited sample size in SAGE is what prompted our analysis of GEUVADIS. One could argue that GEUVADIS sample sizes are also inadequate for our purposes; this is precisely the impetus behind the simulations in our manuscript. We simulated 1000 samples per population, roughly corresponding to the size of PrediXcan DGN or MESA_ALL training sets, thereby assuaging any concerns about realistic population sizes.

Overall, we find the same issues in cross-population prediction as we move to progressively larger sample sizes, from SAGE to GEUVADIS to our simulations. Consequently, we do not feel that we have limited our message by including the analysis of SAGE samples, especially since this analysis is arguably closest to reality for research groups interested in TWAS.

Nevertheless, we want to ensure that readers understand that we progress towards larger sample sizes in the paper. To that end, we have added numerous caveats about sample size in the manuscript. Halfway through paragraph 6 of the Introduction, we now state:

"To tease apart cross-population prediction quality, we turn to GEUVADIS and the 1000 Genomes Project datasets, which includes multiple populations each with more samples than our SAGE cohort.[4,43,44]"

Towards the end of the same paragraph, we now state:

"Finally, to understand the consequences of eQTL architecture on TWAS, we use existing 1000 Genomes data to simulate large samples of two ancestral populations and an admixed population…"

The first paragraph of Results subsection "Cross-population prediction quality declines with increasing genetic distance" now reads:

"…interval to tissue collection (for GTEx).[48–50] The small sample size of our SAGE cohort (n = 39) limits our ability to account for these possible confounders."

In the first paragraph of the Results subsection "Admixture influences cross-population gene expression prediction quality under known eQTL architecture", we explicitly state the simulation sample size, which was previously only mentioned in the Methods:

"We simulated n = 1000 samples for each population, a much larger sample than what is available in GEUVADIS and comparable to the training sample size of DGN or MESA_ALL PrediXcan models."

In the first paragraph of the Discussion, we now state:

"Our investigation with the GEUVADIS dataset[43] offered us a more homogenous environment and larger sample size in which to train and test gene expression prediction models."

In addition to sample size, other confounders like age, population structure, and hidden confounders could also affect performance in SAGE. In the methods, you state that you followed the GTEx v6p eQTL QC pipeline, but details about how many PEER factors, genotypic PCs, etc. were used in your SAGE analyses are needed, especially given n=39 is much smaller than any GTEx eQTL tissue.

The requested details have been added to the Methods section under subsection "Genotype and RNA-Seq data". We adjusted for 3 genotype PCs and 15 PEER factors as specified in the GTEx v6p documentation.

We are aware of possible confounding by age since SAGE is a pediatric cohort, while GTEx contains adults. We stated this limitation in the Discussion:

"Certainly, SAGE differs in important ways from GTEx, DGN, and MESA: SAGE is a pediatric asthma case-control cohort study in African-American children, so we cannot rule out technical heterogeneity introduced by differences in age, study design, and ethnicity."

For the sake of comparability with existing PrediXcan models, we did not adjust expression values for age. We chose to ensure comparable models over correcting for possible age-related heterogeneity (if it exists), as adding unnecessary covariates is unwise in light of our small sample size. If age indeed affects expression prediction, then existing PrediXcan models are structurally biased towards older ages given the specifics of the available studies (e.g. GTEx, MESA, DGN).

By using just one small validation cohort in the first section of your paper (SAGE), key population performance differences among PredictDB models may be missed. I suggest using GEUVADIS populations as validation cohorts of PredictDB models to see if larger sample sizes reveal the expected differences among populations.

Our coauthors previously published this finding (reference #45 in our manuscript). Similar to our analyses here, Mikhaylova and Thornton find that PredictDB models behave differently on the constituent GEVUADIS populations. We remarked on it in the Introduction halfway through paragraph 6:

"However, recent analyses suggest that GTEx and DGN PrediXcan models behave differently on the constituent populations in GEUVADIS.[45]"

In light of the reviewer's comment, we believe that the results from Mikhaylova and Thornton are worth highlighting again. To that end, we have added another sentence to the 3rd paragraph of the Discussion:

“Our results parallel prior evidence [45] that PredictDB models themselves do not predict as well as expected into GEUVADIS despite controlling for tissue type, strongly suggesting that our observations about PrediXcan predictions in SAGE could hold true in other datasets.”

The GEUVADIS results as presented are stronger and a useful demonstration of the cross-population prediction problem observed by others (Mogil et al. 2018, Mikhaylova et al. 2019). The GEUVADIS prediction models built by the authors would be useful to the community and should be made publicly available. While summary stats (R2, rho) were available at https://ucsf.box.com/v/sage-geuvadis-predixcan, the prediction models (i.e. SNP weights per gene) were not included.

All SNP prediction weights have been placed in a Box folder corresponding to the link in the manuscript:

https://ucsf.box.com/v/sage-geuvadis-predixcan

The simulations are thorough and well-presented, and the portion of this paper that goes beyond previous studies. Simulated population models with non-identical eQTLs showed patterns similar to real-world data. The authors call for more diverse sampling in transcriptome studies is timely and necessary in order better compare eQTL architecture among populations.

We thank the reviewer for these encouraging remarks.

Minor:
1. Line 322. Change “different” to “differences”.

Thank you for catching this typographic error. We have corrected it in the manuscript.

2. Line 404. Add “in” to “predicted these populations”.

Thank you for noting this error. This change is now reflected in the manuscript.

3. I think the last sentence fragment in Figure 5 legend should be deleted.

Reviewer #2 also noted this error. The fragment was from a prior version of the figure and is no longer pertinent. Thanks to the reviewers’ careful reading of the figure caption, we have removed the fragment from the caption.

4. Suppl. Fig. 5. Wide bar in the AA 50% circle should be yellow (shared), not red.

In our revised version this corresponds to Supp Fig 19, and it has been changed accordingly.

Reviewer #2: Cross population prediction of gene expression is an incredibly timely subject given that the bulk of match genotype-gene expression data has been done in European ancestry individuals and is use to predict gene expression in diverse population, without complete understanding of the disadvantages or potential errors that may occur. This paper highlights the need to create prediction models in populations

that reflect the ancestry of genotypes used to impute. The authors have used both real and simulated data to make this case, and overall, I feel that paper is useful to the human genetic community. But the manuscript suffers from errors in data visualization that distract from the message and leave the reader confused as to which is the true representation of the data. Below are outline comments to improve and correct these errors.

Major Comments.

1. The number of gene models available in each weighted set would be highly dependent on the criteria used to identify these gene as "well predicted". As an example, depending on the R2 threshold the MESA_All set could have anywhere from 6896 gene model to 1336 gene models (as per the paper). The authors need to add details to the methods on what criteria was used to get to the final list of gene model that are compared. Where these thresholds the same for all weighed sets?

The reviewer makes the important observation that we are limited by existing prediction model sets in PredictDB that range in the number of genes covered. These numbers are given to researchers by PredictDB and are mostly dictated by training sample size, data quality, and other criteria. Furthermore, we are confused where the reviewer obtained the numbers 6896 and 1336, as they do not appear in the manuscript.

We clearly show all thresholding criteria in Supp Table 1 and include the number of gene models from each prediction weight set that meet each threshold. The threshold criteria were the same for all weight sets. Importantly we did not explicitly filter by R^2 for Supp Table 1; rather, we applied a filter for positive correlation between predictions and measurements, as significant negative correlations are hard to interpret.

We suspect that the reviewer is perhaps referring to the analysis behind Figure 3, where we focused on genes from GTEx v7. In that case, we state in the final paragraph of Results subsection "Concordance of measured gene expression and PrediXcan predictions is lower than expected" that we filtered genes with test R2 in GTEx v7 where R2 < 0.2. Our choice of R2 is intended roughly to capture the better half of predictions from DGN; see Figure 3 from Gamazon et al. (2015), which shows that mean R2 in DGN is 0.137. We have added text to the 4th paragraph of Results subsection "Concordance of measured gene expression and PrediXcan predictions is lower than expected" justifying our choice of R2:

> *"Our choice of R2 is informed by observed R2 between predictions and measurements in DGN (see Figure 3 of [6]) and focuses our analysis on genes predicted better than average."*

2. By using all 11,545 genes in the analysis, it seems like it would bias the estimate downward. Especially since the authors compare these vales to the R2 from the training set which do not include all 11,545 genes in all models. While this point is somewhat answered by the GTExv7 analysis on well predicated genes, this does not address if this is true for the more diverse cohorts like MESA.

The reviewer's skepticism of using all genes is warranted, which is why we also partitioned results for subsets of genes; see Supp Figs 2-8 in the revised version of this manuscript. Our subsequent parsing of genes focuses on better predictions and ensures more apples-to-apples comparisons, assuaging any concerns about bias. By any reasonable measure, PrediXcan models do not seem to offer consistently reliable prediction across any logical subset of genes that is not manually cherrypicked. Cherrypicking genes in unrealistic and impractical because researchers using PrediXcan generally do not have expression data to check the quality of predictions.

Regarding the reviewer's comment about our analysis of GTEx v7 genes with cross-validated R2 > 0.2, we have repeated these analyses using MESA models. Four new figures (Supp Figs 9-12) appear in the text with cross-validated testing R2>0.2, one for each MESA prediction model set. The final sentence of the 4th paragraph of

Results subsection "Concordance of measured gene expression and PrediXcan predictions is lower than expected" now reads:

"We see a similar trend with MESA models (Supplementary Figures 9-12), in which R2 in SAGE is consistently much lower (mean R2 0.026 – 0.030) than test R2 from each prediction weight set (test R2 0.373 – 0.392)."

3. The box and whiskers plot are not particularly informative to show the difference in R2 between the weighted sets as highlighted in the results. While I understand the want to graph the test R2 with the actual R2, I think it may be easier to see when graphed separately.

The requested plots appear as Supp Figs 2, 6, and 8, with appropriate references in the Results section.

We remark that the purpose of Figure 1 is to show the overall discrepancy between R2 in the PrediXcan testing sets and actual R2 between predictions and measurements in SAGE. Among SAGE, the R2 distributions do not vary appreciably. This is a point which we feel was not sufficiently stressed in the text, so we have modified the 2nd paragraph of Results subsection "Concordance of measured gene expression and PrediXcan predictions is lower than expected":

"The highest mean R2 of 0.0298 was observed in MESA_AFA, while the lowest was observed in MESA_ALL (R2 = 0.0250), suggesting little appreciable difference in prediction quality between prediction weight sets."

Note that R2 obscures the direction of correlation, which is why we mostly dwell on (signed) correlations in the text; we use R2 to compare to previous work. We stated this in the text:

"As we are primarily interested in describing the relationship between predicted outcome and real outcome, we prefer Spearman's ρ to describe correlations, while for determining prediction accuracy, we use the standard regression R2, corresponding to the squared Pearson correlation, to facilitate comparisons to prior work."

4. As a follow up to comment 2, could the negative correlation described in results (lines 189 – 195) be due to the inclusion of all 11K genes as oppose to those well predicted by each model. Would this still be the case if the authors restricted to just those genes listed in Supplementary Table 1?

The bias that the reviewer describes could possibly exist. But we show in Supp Figs 4-8 that the trends from Figures 1 and 2 still hold true for the 273 genes in common to all weight sets and the 39 of those with nonnegative correlation. These are apples-to-apples comparisons over the same genes and are therefore not biased by the number of available gene models in each prediction weight set.

To ensure that readers are aware of these results, the caption of Supp Table 1 now points readers to Supp Figs 4-8:

"The intersection of genes with both predictions and measurements in SAGE across all seven weight sets is 273 (see Supplementary Figures 4-6), of which 39 produce predictions positively correlated to data in all comparisons (see Supplementary Figures 7 and 8)."

5. The results for the 564 genes that were trained in all GEUVADIS data should be presented similarly to the larger gene set (lines 241-247). Specifically, the author should highlight how the models trained on EUR fared in AFR and vise-versa as oppose to just giving ranges of the R2 across all comparisons.

In response to the reviewer's comment, we have added a substantial amount of text to the second paragraph of Results subsection "Cross-population prediction quality declines with increasing genetic distance":

"A comparison of the 564 genes in common across all train-test scenarios (Table 2) yields a subset of genes with potentially more consistent gene expression levels. In this case involving better-predicted genes, we see that prediction quality between the European groups improves noticeably (p-value ~ 0, Dunn test). Among European training sets, the lowest R2 is 0.183 for EUR278 predicting into EUR278. R2 increases to 0.201 (EUR373 to EUR373) and attains its maximum at 0.216 (EUR278 to FIN), possibly a consequence of diminished haplotypic diversity from Finnish population bottlenecking as mentioned previously. In contrast, R2 between Europeans and Africans ranges from 0.095 (AFR to EUR373) to 0.147 (EUR373 to AFR), a significant improvement (p-value < 7.07 x 10-22, Dunn test) that nonetheless highlights a continental gap in prediction performance. AFR predicts better into FIN (R2 = 0.111) than the other European populations (R2 = 0.095 – 0.096), similar to what we observe with predictions from EUR373 into FIN. But AFR predicts better into itself (R2 = 0.130) than to other populations; similarly, European predictions into AFR are noticeably lower (R2 = 0.141 – 0.147) than into other European populations (R2 = 0.183 – 0.216). In general, populations seem to predict better into themselves, and less well into other populations."

6. I would be helpful to know the population sizes for each on the subpopulations presented on table 3 and 4, unless all of these are 89 as stated in line 252

The caption for Table 3 says:

"All populations were subsampled to N = 89 individuals."

We have added text to the caption for Table 4 to clarify that the results in Table 4 are also from 89 individuals per population:

"…all 25 train-test scenarios. As in Table 3, all populations were subsampled to n = 89 subjects."

7. I am a bit confused on the simulation in which the admixed population inherits causal eQTLs from the parental populations. The explanation in the results states that when k=10 and if only 50% of the alleles are shared, then the AA would have 15 causal eQTLs (5 from CEU, 5 from YRI and 5 shared). So, wouldn't that be 15 causal eQTLs not 10? Also, would it not make more sense use all 5 shared and for the remaining 5 use 80% of the YRI specific and 20% of the CEU specific? While the authors explain the effect of this in the result it is hard to interpret how the simulation would reflect actual admixture.

We have updated the text in the Results subsection "Admixture influences cross-population gene expression prediction quality under known eQTL architecture" to clarify that the choice of *k* causal eQTLs refers to the ancestral populations. The first paragraph now reads:

"We simulate eQTL architectures under an additive model of size k causal alleles (k = 1, 10, 20, and 40) in the ancestral populations (CEU and YRI)…"

The third paragraph now reads:

"…for k = 10 causal eQTLs in the ancestral populations (CEU and YRI)."

Regarding our choice of eQTL architecture, we originally simulated eQTL architectures with 10 eQTLs per population where AA inherits eQTLs in proportion to admixture, precisely as the reviewer suggests. We considered whether this simulation choice was reasonable and opted for the simulation design presented here, under an assumption of varying ancestry-specific effects on each haplotype. We assert that allowing AA to inherit all eQTLs -- and therefore potentially yielding a higher number of causal eQTLs vs. CEU and YRI – is a more biologically plausible model of eQTL architecture, and is consistent with other investigations into genomic architecture in heterogeneous populations, particularly Martin et al. 2017. This simulation assumption is also consistent with the behavior of cis-eQTLs as acting in a haplotype-specific manner. We do note in the manuscript that, when ancestry-specific eQTLs exist, this regime inevitably results in a greater number of relevant eQTLs in the admixed population. All of these assumptions have tradeoffs: under the reviewer's model, this would result in something much higher than the intended 50% shared eQTL architecture, given the preponderance of African ancestry in simulated African Americans.

8. I am confused by Figure 6 as the text and the figure do not seem to match. The authors states," For an architecture with no shared eQTLs, power between CEU to YRI is 0, while power is higher for CEU to AA (0.25) and YRI to AA (0.30)." But the bar chart shows CEU to AA and YRI to AA both to be close to identical at around 0.8.

Thank you for alerting us to this discrepancy. We have updated the text to reflect the numbers in Figure 6.

9. The matching of numbers reported in results to the figures continues on Figure 7 – though admittedly closer. The authors wrote, "For example, when h2 = 0.20 and gene expression is predicted from AD to CEU, power at 0% YRI admixture is 0.56 (95% CI: 0.462 – 0.658) and declines linearly with increasing YRI admixture; at 100% YRI, statistical power for AD to CEU is 0.46 (95% CI: 0.362 - 0.558)." but the line depicted is clearly at 0.5 when the YRI admixture is at 100%. The author need to thoroughly examine all figure to ensure they are depicting what is written in the text.

We thank the reviewer for carefully analyzing Figure 7, but in this case the numbers reflect the average for that specific simulation scenario, whereas Figure 7 shows the linear fits to allow the reader to compare and contrast each scenario. We kept this figure demonstrating the linear trends in the main text. However, based on the reviewer's observation, we have incorporated into the supplement three tables with raw estimates and confidence intervals. The main text now reflects the fact that Figure 7 only shows the linear trends beginning at line 420 of the revised manuscript:

"The phenotypes were simulated at effect sizes β = 0.005, 0.01, and 0.025, and environmental variance σ2 = 0.01, corresponding to heritability h2 = 0.06, 0.20, and 0.58, respectively. To compare and contrast across each train and test scenario, we plot the overall trends of performance in Figure 7, and provide the exact mean power estimates and 95% confidence intervals for each scenario in Supplementary Tables 10-12"

The caption of Figure 7 now concludes with a line pointing interested readers to the new Supp Tables 10-12:

"Raw power estimates and 95% confidence intervals are listed in Supplementary Tables 10-12"

Minor comments:

1. In the simulated gene expression, the author chose cis-heritability of 0.15. Is this the average across all tissues in GTEx, or only LCL, as this may be tissue specific?

This number is chosen to match the h^2 computed for whole blood RNA in Figure 3 of Gamazon et al. 2015. (https://www.ncbi.nlm.nih.gov/pubmed/26258848). The rationale behind choosing h^2 = 0.15 was merely to provide a realistic heritability level consistent with other investigations of eQTL architecture in GTEx and other datasets. We note that our SAGE RNA-Seq data also come from whole blood, so the h^2 used in our simulations is reasonably meaningful for the original observed issue with prediction quality in SAGE.

At this juncture, we also note that the figure in Gamazon et al. (2015) used **DGN** data, not **GTEx** as we stated in the text. We apologize for this oversight and have corrected the text accordingly. We also point the reader back to Gamazon et al. (2015).

The revised text reads:

"…expression phenotype with cis-heritability h^2 = 0.15 (recapitulating the average h^2 in DGN whole blood RNA-Seq data [6])"

2. Why did the authors simulate the AA haplotypes as oppose to use the Hapmap ASW data and simulate haplotypes for that data?

The simulations in this manuscript are meant to illustrate gene expression prediction under two-way admixture. The AA haplotypes are forward-simulated from CEU and YRI for two reasons. Firstly, we can explicitly track the origins of each haplotype with full confidence. Secondly, we can correctly control ancestral proportions of CEU and YRI contributions to our simulated AA population. Using ASW haplotypes in the simulation could introduce inferential uncertainty about the ancestral origin of each haplotype. In addition, HAPGEN2 is not designed to generate realistic haplotypes from an admixed reference panel such as ASW.

3. 2 gene were removed from the TWAS analysis because they had no SNPs in the simulated data. I think this mean there were no SNPs within 2Mb of these genes. This strikes the reviewer as very odd. What is the explanation for this?

The data are taken from the HapMap3 example datasets included with IMPUTE/HAPGEN2:

https://mathgen.stats.ox.ac.uk/impute/impute_v1.html#Using_IMPUTE_with_the_HapMap_Data

Based on the reviewer's question, we re-confirmed in this data freeze of HapMap3 that there are no segregating variants around these genes to provide any signal. To clarify this point, we modified the text in the Methods section to reflect the reviewer's intuition:

"We removed two genes, PPP6R2 and MOV10L1, that spanned no polymorphic markers within 2 megabases of their start and end positions in the HapMap3 dataset, resulting in 98 gene models used for analysis."

4. It would be useful to the reader to add the github repositories that contain the PredictDB models that were used in the paper.

A hyperlink to PredictDB already appears under the section "Online Resources", along with links to our own code and results, as well as public data sources such as GTEx, DGN, and GEUVADIS. Note that PredictDB uses Github to organize source code, but the prediction models themselves are posted on predictdb.org. As we previously described, we have set up a Box account for releasing our own models.

5. Gene numbers do not agree between the results and the methods. As an example, the methods states that 10,161 gene were found in at least one weighed set. But in the result the number is 11,545.

Thank you for catching this oversight. The correct number is 11,545 genes total. The Methods section now reflects the correct number.

6. The authors point out the least negative correlation was seen with MESA_AFHI and the most with MESA_AFA, but the number is the results are not shown on Supplementary Table 1

Thank you for this as well. The numbers in the text have been updated and now match Supp Table 1.

The third paragraph of Results subsection "Concordance of measured gene expression and PrediXcan predictions is lower than expected" now reads:

"The least negative mean correlation across prediction weight sets was observed in GTEx v6p (-0.0044), while the most negative mean correlation (-0.0204) was observed with MESA_AFA (MESA African Americans, Supplementary Table 1). … While there are some fluctuations in prediction accuracy, the fact that correlations vary from -0.0204 to -0.0044 indicates that no prediction weight set produces practically meaningfully better correlations to data than the others."

7. "Utahans" is misspelled on line 217

We have followed the convention from utah.gov, where the state government uses the demonym "Utahns."

8. Figure 5 is incomplete. The ledged reads, "A dotted red line at h2 = 0.95 marks the power values shown in". Shouldn't this be at 0.205 (assuming this is related to Figure 6) also there is no red line.

Reviewer #1 also remarked on this. We thank both reviewers for highlighting this error. The reviewer is correct that Figures 5 and 6 are related – Figure 6 presents a cross-section of the panels of Figure 5 at $h^2 = 0.205$. The sentence fragment was from a previous version of the figure and has been removed.

Reviewer #3: In this manuscript, the authors describe a impressive set of analyses on the portability of gene expression QTLs (eQTLs) across diverse ancestries. They then extend this work to look at Transcriptome-Wide Association Studies (TWAS), and evaluate the extent of portability of these models across ancestry groups.

In the case of complex traits, we have larger sample sizes and are often discouraged by the losses in portability there, but this paper clearly makes the important point that the situation is even more discouraging for gene expression. The paper is well written and all analyses seem thoughtfull laid out. In addition, there has been momentous effort to make the data and software freely available, which should be commended. That being said, there are a few limitations to the current analyses, detailed below.

We thank the reviewer for their encouraging remarks about the manuscript. Our responses to the reviewer's concerns are given below.

1) The data come from myriad studies of: whole blood, PBMCs, monocytes, and LCLs. There is not a direct comparison between sample types, despite the availability of GTEx models trained on both LCLs and whole blood. Adding such a comparison of trained LCL/WB models would help researchers apply the results. (The

lack of direct comparison to monocytes is understandable given the very preliminary nature of the MESA whole blood RNA-seq data generated as part of TOPMed, and the lack of existing TWAS models. As such, lack of monocyte comparison I don't think is a concern.)

Correctly matching tissues is indeed important when predicting gene expression, but we assure the reviewer that we have given the most direct comparisons possible. We take MESA monocyte models as-is for reasons that the reviewer mentions. Comparisons with GTEx and DGN use prediction models trained in the same tissue (whole blood) that we have in SAGE. Using GTEx LCL models in SAGE fails to match tissue types and is unlikely to provide additional insight into cross-population generalizability of the models. Realistic cross-tissue analyses require MultiXcan (see Barbeira et al. 2019, https://doi.org/10.1371/journal.pgen.1007889) and are beyond the scope of this manuscript.

The concern about LCLs presumably concerns GEUVADIS since GEUVADIS expression data were taken from LCLs. But we specifically state in the final paragraph of the Introduction:

> *"We train, test, and validate predictive models wholly within GEUVADIS with a nested cross-validation scheme."*

We previously tested PrediXcan models in GEUVADIS in Mikhaylova and Thornton (2019) (reference #43 in our paper). That analysis of prediction quality into GEUVADIS included both whole blood and LCL prediction models. Incidentally, we observed similar cross-population issues to what we found here, regardless of tissue matching.

2) GTEx, particularly in v8, has a substantial number of African-American and Hispanic participants with LCLs and Whole Blood. If possible, these should be included as a separate group for evaluation, to test whether the claim regarding SAGE and MESA_AFA is generalizable to other datasets. There are numerous different factors which might be contributing to the lack of reproducibility (e.g. WGS vs genotyping arrays; age effects; RNA isolation and sequencing protocols; source material; etc) and including GTEx Whole Blood and LCL expression in HIS and AFA individuals as evaluation sets would enable direct evaluation of some of these factors.

We do agree with the reviewer that disentangling potential technical heterogeneity would help understand these models, but providing a comprehensive investigation of potential sources is beyond the scope of this manuscript. We used PrediXcan whole blood models to match the tissue (whole blood) with our SAGE data, as an example of a real-world scenario and potential application setting. We note that the full SAGE study has nearly 1,800 participants with whole-genome genotype data (of which the 39 with RNA-seq data are a subset) and would be an ideal candidate for an application such as this. We used the MESA monocyte models for the reasons that the reviewer noted in the previous comment. We did not use LCL prediction models in SAGE since the whole blood ones are appropriate, and consistent with the reviewer's comments LCL models should be highly different.

Regarding the evaluation of other GTEx groups, we note that GTEx does not identify study subjects as Hispanic or Latinx, so presumably the reviewer is interested in behavior of PrediXcan models tested in African Americans from GTEx v8. We strongly urge caution when making comparisons between models trained or tested in different versions of GTEx. GTEx v8 uses a separate imputed variant set. While it would be interesting to test in GTEx v8 African Americans, the comparison is not trivial. Furthermore, our analysis with GEUVADIS was intended to test the scenario of limited technical heterogeneity. Our simulations derived from the well-characterized GEUVADIS samples still yielded substantial evidence of impaired cross-population generalizability, as well as some guidance on when they do (namely, when the eQTLs are the same across populations).

3) It would be nice to know whether the 564 genes (Table 2), 142 genes (Table 4), or 273 genes (Supplementary Table 1) are representative of the whole transcriptome. A simple violin plot of the expression distribution of these genes and other genes, within each population, would suffice. It might be helpful to see their Test R^2 estimates as well, but I don't think that's critical.

The requested violin plots for SAGE (using GTEx) and GEUVADIS (broken down by population) have been added to the supplement (new Supp Figs #1, 3, 5, 13, 14, 15, 16) and are now referenced in the appropriate paragraphs of the Results section.

In Supp Fig 1 we use log-transformed TPM values from GTEx to show differences in baseline gene expression between all GTEx v7 genes versus the 273- and 39-gene subsets discussed in the manuscript. Supp Figs 3 and 5 show distributional summaries of R2 in SAGE versus test R2 from PredictDB using a "transcriptome-wide" set of 11,545 genes (Supp Fig 3) and the 273-gene subset (Supp Fig 5).

For Supp Figs 13-16 we plotted distributions of testing R2 broken down by train-test scenario; each supplementary figure shows a different subset of genes over the same data. In general, the subsets have genes that are expressed slightly better than average.

We note that our original boxplots show that the distributions of R2 cluster around 0 for any reasonably well predicted set of genes. The story is slightly different for the training R2 from each repository, since the 273 genes in common to all repositories are slightly better predicted on average during training. Nevertheless, these 273 genes are not well predicted on average in SAGE.

4) Regarding the TWAS simulation: While the simulation itself only used 100 genes, I think that readers would also be interested in understanding the power were a whole genome expression panel were used. Is it possible to recompute significant thresholds and provide these "transcriptome-wide" significance estimates as well? It would also be useful to know what the expected variance explained by the genes is -- my understanding, for beta = 1, is that you are adding N(0, 0.1^2) noise still, so heritability should be very high? (It's a bit unclear whether the h^2 = 0.15 applies to the TWAS simulation as well, and lines 143-149 of simulate_twas_sge.R seem to indicate that h^2 isn't used.)

If the reviewer is interested in extending this script for power calculations for whole-genome investigations, the relevant variable to modify in simulate_twas_sge.R for genome-wide power analysis is "ngenes" on line 170, that can be modified along with input sample size. We note that the trends would be expected to be monotonic with those observed in our simulation regime. The simulations use chromosome 22 to allow the simulations to act on a single chromosome. Across numerous simulation instantiations, our simulations provide sufficient complexity without needing to extend the approach to multiple chromosomes.

Regarding phenotype heritability in our simulations of TWAS power, we stated in the methods that we use a single noise distribution but vary the effect sizes to cover a range of heritability:

> *"Effect sizes were fixed, and we tested various effect magnitudes β = 1 x 10-5, 5 x 10-5, 1 x 10-4, …, 1 x 10-1, 5 x 10-1, 1. The environmental noise ε was drawn from an N(0,0.1^2) distribution. Consequently, phenotypes therefore only varied with the expression measures from G."*

This yields a spectrum of phenotypic heritability or proportion of phenotype variance explained (PVE) as in x-axis of Figure 5 and the panel labels of Figure 7. We have updated the Methods text to clarify this:

"Effect sizes were fixed, and we tested various effect magnitudes β = 1 x 10-5, 5 x 10-5, 1 x 10-4, …, 1 x 10-1, 5 x 10-1, 1, yielding a spectrum of phenotype heritability explained by gene expression. …"

In contrast, the genetic heritability of expression is set to 0.15, but that is not the same as the TWAS phenotype of interest. The h2 lines in our code are placeholders from when we simulated phenotypes differently and are commented out because they are no longer used. In our current simulation scheme, h2 is calculated from the varied effects and noise.

Points of clarification or minor analysis:

5) Given that the SAGE participants were ascertained on the basis of rs28450894, some validation that effect sizes are not out of line at this locus is important to understanding the results. How close are the 273 genes to this SNP, and does this SNP (or a close LD partner) have non-zero weight in any models? It does seem rather unlikely that this SNP (or bronchodilator status in general) are driving the lack of signal, though, so simply acknowledging this and noting these distances and weights is sufficient in my opinion.

We have added two supplementary tables (Supp Tables 3 and 4) and a new paragraph arguing that no obvious ascertainment bias exists in our analysis:

"Since SAGE data were ascertained on the basis of rs28450894 and by extension gene NFKB1 [39], we checked if results were biased by ascertainment. Among the 273 genes in common to all weight sets, only one gene model, SLC39A8, lay within 1 megabase in either direction of rs28450894 on chromosome 4. Only two of the SNP predictors for SLC39A8 showed more than moderate linkage disequilibrium (R2 > 0.2) with rs28450894: SNP rs72696152 (MESA_ALL, R2 = 0.675) and rs4648011 (DGN, R2 = 0.262) (Supplementary Table 3). However, the resulting prediction quality were close to 0 like the remaining 272 genes, as the linear model R2 for SLC39A8 ranged from 0.0007 (GTEx v7) to 0.0102 (GTEx v6p), indicating no obvious bias away from 0 (Supplementary Table 4)."

6) It appears that there is now a HIS weight set for MESA available on predictdb.org -- I would suggest either including, or rewording "all four" (line 153) to "four of the".

We thank the reviewer for alerting us as we were not aware of this development. We have edited line 153 (now line 154 in the revised manuscript) to say "four of the MESA monocyte weight sets".

7) Are you powered (or is it possible to obtain) to estimate the heritability of the 273 genes in each population? Evaluating whether there is a relationship between R^2 and h^2 would be valuable. Currently, the Test R^2 is the comparison group, which should still be included but is perhaps underpowered and should not be considered a true upper bound. In particular, it would be nice to know if h^2 is different in AFA than in EUR. At the very least, showing a scatterplot of Test R^2 in EUR vs AFA in MESA/GEUVADIS of the different gene sets would be helpful.

The relationship between genetic heritability and R2 between predictions and measurements was established in Figure 3 of Gamazon et al (2015) with DGN. However, DGN has almost 1000 individuals. None of the real datasets in this manuscript (SAGE, n=39; GEUVADIS AFR, n=89; GEUVADIS EUR, n=373) are sufficiently powered to provide reliable heritability estimates, or even test for a significant difference from 0, using GCTA and the method for correcting h2 confidence intervals from FIESTA (Schweiger et al. 2018).

Originally we did investigate h2 vs. R2 for GEUVADIS EUR, but unfortunately that dataset is also limited by sample size.

As we do not have individual-level access to MESA we cannot perform the suggested test-R2 analysis, but the performance of these models was investigated by Mogil et al. 2018 (reference #38 in our manuscript).

8) Adding to Table 1 the number of individuals in the train and test populations, as well as the number of genes with positive correlation, would help with direct interpretation of Table 1 and Table 2. In addition to the R^2 measures, perhaps a direct test of e.g. test statistic inflation be more interpretable?

Some of the names used in Tables 1 and 2 state the sample size (EUR373 and EUR278). The remainder have sample sizes stated in the text (FIN = 95, AFR/YRI = 89). We have added the sample sizes for each sample in the captions of Tables 1 and 2.

For Table 1, the number of genes used in each train-test scenario varies; some training sets produced gene models that others did not, driven in part by different training sample sizes. This mimics precisely the reality of existing PrediXcan models, since each repository has a different number of gene models and a different training sample size. Consequently, we did not mention the number of genes compared. To address this, we have added text in the caption to point interested readers to Supp Table 5:

"The number of genes analyzed in each scenario varied in each case; see Supplementary Table 5."

Unlike Table 1, the comparisons in Table 2 were over the same genes. Thus, Table 2 provides an apples-to-apples comparison across genes (but not against *sample sizes*; this motivates the analysis behind Tables 3 and 4). We modified text within Table 2 to repeat from the caption and state the number of genes represented in each table.

We understand the reviewer's question about test statistics, and were the models more generalizable to the scenarios in Tables 1 and 2, this may be more illuminating. However, in light of the issues seen during our test, we believe that displaying the correlations is a more appropriate description and that there would be limited test statistic inflation.

7) What does it mean for CEU and YRI to have 0.0 shared ancestry? I see the simulation used haplotypes from CEU, YRI, or a mix thereof, but these sets of haplotypes can overlap. Some measure of haplotype sharing would be appreciated (or just stating the simulation proportion mixing directly, without making a population-level claim).

For the purposes of our simulation, CEU and YRI are distinct populations. The reviewer is correct to note that the two populations can still share haplotypes, but this would reflect the reality of deep shared ancestry that can exist, even within recently admixed populations. Given much previous research on this topic, we note that CEU and YRI represent reasonable proxies for the ancestral diversity present in modern African Americans (in particular, see Baharian et al. 2016, reference #56 in our manuscript), so whatever haplotype sharing exists here could also be present in real populations.

The places where we did not change the text are the title of Results subsection "Power to detect associations declines with decreasing shared ancestry" and the first sentence of that section.

We did change the caption of Figure 6. We replaced all instances of "shared ancestry" with the more precise phrasing "admixture proportion" as the reviewer suggests. Unlike the subsection header, here we explicitly refer to populations CEU, YRI, and the simulated admixed AA.

8) Do you have a sense of why AA->AA is so much better than CEU->CEU or YRI->YRI in Supplemental Figure 9? Should I be interpreting this in the context of total haplotypic diversity in AA?

The figure that the reviewer references, Supp Fig 9, is now Supp Fig 23 in this revision. The results under 0% or 50% shared eQTL from AA to AA are better than CEU or YRI into themselves as a consequence of the eQTL architecture (see Supp Fig 19 in revised manuscript). As we phrased it, the term "shared eQTL architectures" refers to how many eQTLs were shared by the ancestral populations (CEU and YRI). Since AA inherits all eQTLs, the result is that AA typically has more eQTLs than either CEU or YRI. This drives differences in the t-statistics, and therefore influences p-values and power. We note that differences in t-statistics in Supp Fig 23 exist between populations even when all eQTLs are the same across populations, which is a function of haplotype sharing between AA and its ancestral populations CEU and YRI, as well as the diminished haplotype sharing between CEU and YRI.

9) It seems from Supp Table 3 like the FIN testing population, trained in AFR, does substantially better than the other EUR test populations. Any idea why that might be?

If we understand correctly, the reviewer is asking why AFR predicts better into FIN (R2 = 0.039) than into EUR373 (all 4 EUR pops, R2 = 0.029) or EUR278 (3 non-Finnish EUR pops, R2 = 0.030).

The simplest explanation is that FIN is a single population, while EUR373 and EUR278 are composed of multiple populations (EUR373 = CEU + GBR + TSI + FIN; EUR278 = CEU + GBR + TSI). Consequently, prediction into a heterogenous test set such as EUR373 or EUR278 may be harder than prediction into the more homogeneous test set FIN.

A potential explanation is that the population bottlenecking in Finnish demographic history has reduced haplotypic diversity in FIN, while the population history of YRI has yielded more genetic diversity in YRI. This means that YRI is capable of predicting reasonably well into FIN, whereas FIN lacks sufficient genetic diversity to predict as effectively into YRI. A similar story could be told about the relationship between YRI and each of the four EUR populations, though we note that the Finnish reflect more isolation than the other three EUR pops. This latter explanation is somewhat supported by Table 4, in which YRI predicts better into EUR pops than EUR pops do into YRI. However, a full investigation of this would need to be completed in future research.

10) For simulated gene expression that went into the TWAS, was there a minor allele frequency cutoff in choosing causal eQTLs? And was this matched across populations?

Yes, the minimum admissible MAF for eQTLs in our simulations was 5%. The threshold was the same for all populations. Our source code reflects this:

https://github.com/klkeys/sage-geuvadis-predixcan/blob/master/analyses/03_hapmap3-simulation/src/02_test_prediction_models/simulate_crosspopulation_prediction.R

We have added the following text to the Methods subsection "Simulation of gene expression":

"Causal eQTLs were chosen at random among SNPs with at least 5% minor allele frequency. The same 5% minor allele frequency floor was applied to each population."

11) "The comparison of predictive models cannot easily differentiate predictions of 0 (no gene expression) and NA (missing expression) [L570-572]." -- Could you please clarify this statement?

A prediction of no gene expression is different than a missing expression level. The former denotes a gene that is not expressed, while the latter denotes a gene for which we do not know the expression level. However, they both affect mean expression levels; the former biases averages towards 0, while the latter generates a smaller denominator in averages and therefore biases expression levels towards whichever genes were actually measured.

We noted this observation in the manuscript because dealing with mean gene expression does require some care to disentangle these two situations.

Nevertheless, the reviewer's remark does highlight possible reader confusion at this point. We have rephrased the text in Methods to read as follows:

"We applied two additional filters to ensure that gene expression models were suitable for analyses. Firstly, we removed genes that did not have any eQTLs in their predictive models. Secondly, genes where fewer than half of the individuals had nonmissing predictions were removed from further analysis. This latter filter discarded those genes for which expression was not easy to predict across multiple samples."
