# Supplementary material for: On the cross-population generalizability of gene expression prediction models
Source: PLoS Genet. 2020 Aug 14;16(8):e1008927. doi: 10.1371/journal.pgen.1008927 (PMC7449671; doi:10.1371/journal.pgen.1008927)
Supplement: Attachment — Submitted filename: Keys et al Response to Reviewers PLOS Genetics submission 3.pdf [file pgen.1008927.s037.pdf]

Comments to the Authors:

Reviewer #1: The authors have addressed my concerns satisfactorily. The only minor adjustment in the text I suggest is removing/rewording this sentence from the Introduction, line 138: "To our knowledge, nobody has investigated cross-population generalizability of new prediction models generated within GEUVADIS." Fryett et al. very recently (March 2020) published a study in Genetic Epidemiology that did this (https://doi.org/10.1002/gepi.22290). I understand their work was completed in parallel to yours, but I suggest removing this now inaccurate sentence. Doing so will in no way diminish your thorough investigation into the important problem of cross-population portability presented here. Thank you for your thoughtful response to the reviews and be well.

Thank you for alerting us to this reference. We have deleted the sentence as suggested and added a citation to Fryett, Morris, and Cordell (2020) in the previous sentence as reference #46. The section in question (lines 137-139) now reads:

"However, recent analyses suggest that GTEx and DGN PrediXcan models behave differently on the constituent populations in GEUVADIS.[45,46] GEUVADIS provides us an opportunity to investigate predictive models with an experimentally homogeneous dataset…"

Reviewer #3: Thank you for your thoughtful and thorough response piece. I appreciate your inclusion of this additional material and I believe the resulting work is acceptable. A few brief comments:

1) It is worth noting that GTEx does have (self-report/close-relative-report) ethnicity in the ETHNCTY variable:
https://ftp.ncbi.nlm.nih.gov/dbgap/studies/phs000424/phs000424.v8.p2/pheno_variable_summaries/phs000424.v8.pht002742.v8.p2.GTEx_Subject_Phenotypes.var_report.xml
which I note because it might be helpful to include in future analyses. However, while I would still like to see the relative differences due to switching datasets versus switching cell types, I think the data are fine as they are. I would just request the authors make this limitation more clear, and that the predictions observed might change on other datasets from the same cell type versus on different cell types within whole blood.

As comments #1 and #2 are closely related, we provide a complete response to comment #2.

2) In particular, I don't understand the argument that GTEx v8 and GTEx v7 are sufficiently different that they cannot be compared. Were that the case, would SAGE not also be too different to be compared? It might be worth noting cases under which whole blood could be predicted more accurately than in LCLs, e.g.

https://pubmed.ncbi.nlm.nih.gov/19043577/.

For this paper, we focused on matched tissue where possible (whole blood in the case of SAGE), with the notable exception of MESA models, which are only available in monocytes. Our goal was never to test performance of PrediXcan in multiple tissues but rather to investigate the generalizability of Predixcan across diverse populations. Other works, particularly Mikaylova and Thornton (2018) and Fryett, Morris, and Cordell (2020) have done this to varying degrees that are not within the scope of this work.

We appreciate the reviewer's concern about comparing datasets and different versions of GTEx. However, we wish to clarify that we wanted to investigate the performance of different GTEx models within the context of external data such as SAGE. Investigation of GTEx models on internal subsets of left-out GTEx data would be a natural future direction to analyses such like this in additional works.

3) In the abstract: "the amount of shared genotype predictors" is unclear and I would re-word to indicate that this refers to genetic variants included in the model.

Thank you for pointing this out. We have edited the phrase in question that appears in the Author Summary on line 59 to "*proportion of genetic variants shared between population-specific prediction models.*"

4) The following response: "However, in light of the issues seen during our test, we believe that displaying the correlations is a more appropriate description and that there would be limited test statistic inflation." Suggests to me that including the test statistic inflation analysis would aid interpretation of the results. Alternatively, the fraction of FDR-adjusted positive correlations (under a half-normal distribution) could play a similar role. I think that everyone expects the power to be limited, but it is useful to have some measure of error on the R^2 measures. (for instance, with the current rendition it is unclear whether FIN is indeed better predicted than EUR278 with AFR weights)

Results in our Dryad deposition (see comment below about RNA-Seq data release) and described in our data availability will include p-values for every gene in every train-test scenario, consistent with the need for both effect size and significance per model. This provides utility for downstream questions such as that proposed by the Reviewer, where 61% of genes from Table 1 (95% CI [58.9%, 63.2%]) from the AFR training set are better predicted in FIN than in EUR278.

5) Regarding your response to the FIN prediction, it suggests that if heterogeneity is driving differences in prediction, a meta-analysis across populations might be more appropriate.

However I think the point is clear enough as is that such an analysis is likely above and beyond.

Otherwise, I think the manuscript is clear and comprehensive.
We thank the reviewer for the encouraging remarks.

Have all data underlying the figures and results presented in the manuscript been provided? Large-scale datasets should be made available via a public repository as described in the *PLOS Genetics* data availability policy, and numerical data that underlies graphs or summary statistics should be provided in spreadsheet form as supporting information.
Reviewer #1: No: SAGE RNA-Seq data did not appear to be available through dbGaP phs000921.v4.p1, just WGS. Please correct me if I'm wrong or provide an RNA-Seq accession or details on how the RNA data may be accessed.

The RNA-Seq data were not funded by NIH and are therefore not subject to dbGaP data release policy. We have taken the opportunity to move all of our real and simulated data and results from our institutional Box service to the Dryad data repository, where we can manage the data release ourselves. Additionally, Dryad includes a versioning system and furnishes a DOI for our data release, which better conforms to PLOS data sharing policy and aids in both long-term reproducibility and data curation.

For review purposes, the following download link is available:
https://datadryad.org/stash/share/OanyyyoL1zwLNL2Uf7S9pPUvHtltxUYCYXBMLc40k18

Dryad has provided the following DOI for our data and results release:
https://doi.org/10.7272/Q6RN362Z

We have replaced the public Box link (https://ucsf.box.com/v/sage-geuvadis-predixcan) with the aforementioned DOI in the manuscript on line 562. The Box link will remain active as well until Dryad activates the DOI.
